# Behavioral gain following isolation of attention

**DOI:** 10.1038/s41598-021-98670-w

**Published:** 2021-09-29

**Authors:** Grace Edwards, Anna Berestova, Lorella Battelli

**Affiliations:** 1 Center for Neuroscience and Cognitive Systems@UniTn, Istituto Italiano di Tecnologia, Rovereto, Italy; 2 Department of Psychology, Harvard University, Cambridge, MA 02138 USA; 3 Lesley University, 29 Everett St, Cambridge, MA 02138 USA; 4 Berenson-Allen Center for Noninvasive Brain Stimulation and Department of Neurology, Beth Israel Deaconess Medical Center, Harvard Medical School, Boston, MA 02215 USA

**Keywords:** Neuroscience, Psychology

## Abstract

Stable sensory perception is achieved through balanced excitatory-inhibitory interactions of lateralized sensory processing. In real world experience, sensory processing is rarely equal across lateralized processing regions, resulting in continuous rebalancing. Using lateralized attention as a case study, we predicted rebalancing lateralized processing following prolonged spatial attention imbalance could cause a gain in attention in the opposite direction. In neurotypical human adults, we isolated covert attention to one visual field with a 30-min attention-demanding task and found an *increase* in attention in the opposite visual field after manipulation. We suggest a gain in lateralized attention in the previously unattended visual field is due to an overshoot through attention rebalancing. The offline post-manipulation effect is suggestive of long-term potentiation affecting behavior. Our finding of visual field specific attention increase could be critical for the development of clinical rehabilitation for patients with a unilateral lesion and lateralized attention deficits. This proof-of-concept study initiates the examination of overshoot following the release of imbalance in other lateralized control and sensory domains, important in our basic understanding of lateralized processing.

## Introduction

Avoiding hazards while driving, catching a ball, and spotting your friend in a busy restaurant involve careful deployment of lateralized visual, attentional, motor, and tactile processing. Left and right hemispheric regions control right- and left-sided processing of exogenous input and endogenous control, respectively. Here, we examine the outcome of prolonged imbalance in lateralized processing.

Short-term imbalance between right and left lateralized processing causes an inhibitory interaction between lateralized regions, prioritizing processing in the activated hemisphere. For example, left limb tactile stimulation inhibits somatosensory processing of the right limb tactile stimulation^1^, and inhibitory non-invasive brain stimulation to left attention regions increases right hemispheric attention processing^2^. Studies on patients with unilateral lesions provides a behavioral account of severe and ongoing lateralized processing imbalance. Deficits in left visual field attention are typically found in patients with a right frontal-parietal unilateral lesion. Kinsbourne^3^ suggested that attentional neglect of the contralesional visual field results from a combination of the lesion and a hyperactivation of the healthy hemisphere, which further inhibits the lesioned cortex. The hyperactivity of the healthy cortex is hypothesized to be in response to a lack of inhibition from the lesioned cortex^3,4^. However, patient studies are unable to detail how lateralized processing is rebalanced *after* a period of imbalance, which occurs often in the healthy cortex. For example, when driving on the outside lane of a highway, we fixate on the road ahead, but attend to the right visual field continuously for hazards. How does this prolonged imbalance affect subsequent attention processing across the visual field?

Top-down, task-relevant attention is controlled by a large cortico-subcortical network including the dorsal frontal-parietal regions^5^, superior colliculus (SC) in the midbrain, pulvinar nucleus of the thalamus^6^, and cholinergic inputs from the basal forebrain^7^. The dorsal frontal-parietal attention regions span the intraparietal sulcus (IPS), superior parietal lobule (SPL), frontal eye fields (FEF) and dorsolateral prefrontal cortex (PFC) in both the left and right hemisphere^5,8^. fMRI evidence supports that spatial attention to the left and right visual field is lateralized to right and left hemispheres, respectively^9–12^. However, there is evidence the right hemisphere has representation of ipsilateral and contralateral visual fields^13^. It is plausible that rebalance following visual field specific imbalance could be mediated between right and left hemispheric lateralized attention processing regions, or solely within the right hemisphere with representation in both visual fields.

Studies on monocular deprivation (i.e. prolonged covering of one eye) have provided some insight to the product of prolonged imbalanced in sensory processing. Prolonged monocular deprivation increases the processing strength of visual input to the deprived eye, post-deprivation^14–16^. Examination of GABA concentration indicated a reduction of inhibition between the ocular dominance columns of the left and right eye in the early visual cortex^17^. Therefore, with long-lasting sensory imbalance, the commonly found inhibitory interaction between the eyes is muted, resulting in an increased processing of sensory input to the deprived eye. To our knowledge, monocular deprivation is the only other paradigm developed to examine the period after lateralized sensory imbalance. Monocular deprivation imbalances sensory input in visual processing, whereas we intend to record the behavioral impact of an imbalance in lateralized attention processing.

**Figure 1:**
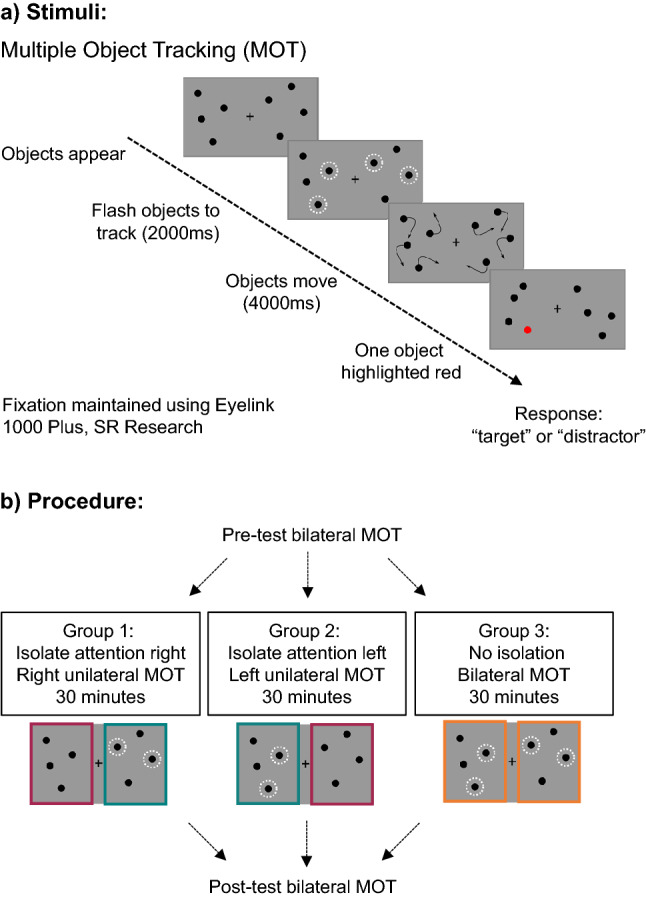
(**a**) Bilateral Multiple Object Tracking (MOT) stimuli. Visual field specific attention measured using bilateral MOT or unilateral MOT (depicted in 1b). Fixation maintained using gaze-contingent programming with Eyelink 1000, SR Research. (**b**) Procedure. Unilateral MOT employed to isolate attention to one visual field for 30 min in Groups 1 and 2. The difference from pre- to post-test bilateral MOT is determined for the Attended Visual Field in teal, Ignored Visual Field in maroon, and in the control visual fields in orange.

Here we determine the impact of prolonged imbalance in lateralized visual attention on subsequent attentional control. Forty-two participants first performed bilateral Multiple Object Tracking (MOT) to obtain a baseline attention performance in the left and right visual fields (Fig. 1a). MOT measures covert attention as participants track target objects in amongst distractor objects in the left and right visual fields separately, whilst maintaining central fixation. Next, we imbalance attention sensitivity by presenting task relevant stimuli to one visual field and task irrelevant stimuli in the other^18^. Participants were randomly assigned to one of three experimental procedures (Fig. 1b): Group (1) performed right unilateral MOT whilst maintaining fixation and ignoring MOT stimuli on the left. This isolated their attention to the right visual field for 30 min. Group (2) performed left unilateral MOT while ignoring the MOT stimuli on the right, isolating their attention left for 30 min. Control Group (3) performed 30 min of bilateral MOT, tracking targets on both left and right. Immediately after the manipulation all participants performed bilateral MOT. Following prolonged attention imbalance, we expected an increase in tracking performance in the ignored visual field for Groups 1 and 2.

## Results

### Isolation manipulation impact on tracking performance

**Figure 2:**
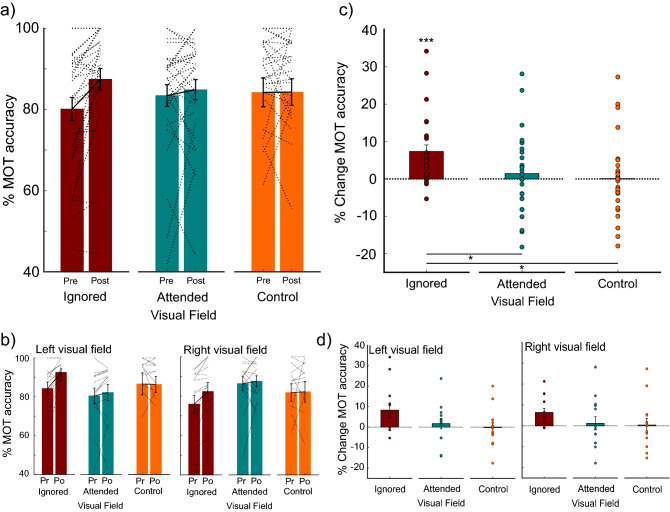
Percentage and percentage change MOT accuracy for pre- and post-manipulation. (**a**) Pre- and post-manipulation percentage correct MOT accuracy collapsed across left and right visual fields for ignored visual field (maroon bars), attended visual field (teal bars) and Control condition (orange bars). Dotted lines represent individual subject data. (**b**) Pre- and post-manipulation separated by visual field. (**c**) Percentage change in MOT accuracy. Asterisks above bars indicate significant difference from pre- to post-manipulation (***p = 0.0001 maroon bars; *p = 0.0205 between Ignored and Control; *p = 0.0439, between Ignored and Attended). (**d**) Data separated by visual field, demonstrating no difference if manipulation is performed in right or left visual field.

We first examined the impact of prolonged tracking in one visual field on modulation of visual field specific attention from pre- to post-isolation. We expected an increase in tracking performance from pre- to post-manipulation in the Ignored Visual Field (maroon outline, Fig. 1b). No tracking performance change was expected for the Attended Visual Field (teal outline, Fig. 1b) or the Control Visual Fields (orange outline, Fig. 1b). We found a main effect of Session (pre- versus post-manipulation; χ^2^(1) = 7.8866, *p* = 0.0050, *glmer*) and no main effect of Visual Field (left versus right; χ^2^(1) = 3.2210, *p* = 0.0727, *glmer*) or Manipulation (Attended Visual Field, Ignored Visual Field, or Control; χ^2^(2) = 1.0618, *p* = 0.5881, *glmer*). We also found no three-way interaction between Session, Visual Field and Manipulation (χ^2^(2) = 1.9270, *p* = 0.3816, *glmer*). However, in examining the two-way interactions, we found an interaction between Manipulation and Session (χ^2^(2) = 9.4680, *p* = 0.0088, *glmer*; Fig. 2a, b). This interaction indicates a significant change in tracking from pre- to post-manipulation as a function of attention isolation, however this interaction was not different across left and right visual fields (Fig. 2b, d). Model comparisons support Manipulation x Session interaction as the best and least complex fit for our data (see Supporting Information).

Collapsing across visual field, we found a significant increase in tracking performance from pre- to post-manipulation for the Ignored Visual Field (Fig. 2c; Maroon bar, estimate = 0.6683, se = 0.164, z = 4.079, *p* = 0.0001, confidence interval (CI) = 0.278, 1.059; *emmeans()* with *adjust “mvt”*). No change in tracking performance was found for the Attended Visual Field (Teal bar, estimate = 0.2052, se = 0.161, z = 1.277, *p* = 0.487, CI =  − 0.173,0.588; *emmeans()* with *adjust “mvt*) or for the Control (Orange bar, estimate = 0.0244, se = 0.190, z = 0.128, *p* = 0.9989, CI =  − 0.334, 1.299; *emmeans()* with *adjust “mvt*). Furthermore, we found a significant difference in tracking between the Ignored Visual Field and the Control (estimate = 0.0727, se = 0.0276, t.ratio = 2.634, *p* = 0.0205, CI = 0.01, 0.135; *emmeans(),* with *adjust “mvt”*) and between the Ignored and Attended Visual Fields (estimate = 0.0590, se = 0.0251, t.ratio = 2.353, *p* = 0.0439, CI = 0.001, 0.117; *emmeans(),* with *adjust “mvt”*). Therefore, the attention isolation manipulation only increased performance in the Ignored Visual Field, significantly more so than in the Attended Visual Field or Control.

Examining reaction time, we found a main effect of Session (pre- versus post-manipulation; χ^2^(1) = 28.2633, *p* < 0.0001, *glmer;* Supplemental Fig. 1) and no main effect of Manipulation (Attended Visual Field, Ignored Visual Field, or Control; χ^2^(2) = 1.2114, *p* = 0.5457, *glmer*). We also found no two-way interaction between Manipulation and Session (χ^2^(2) = 0.8457, *p* = 0.6552, *glmer*). This analysis demonstrates participants became faster at the MOT task, regardless of manipulation.

### Pre-, during-, and post-manipulation tracking performance

**Figure 3:**
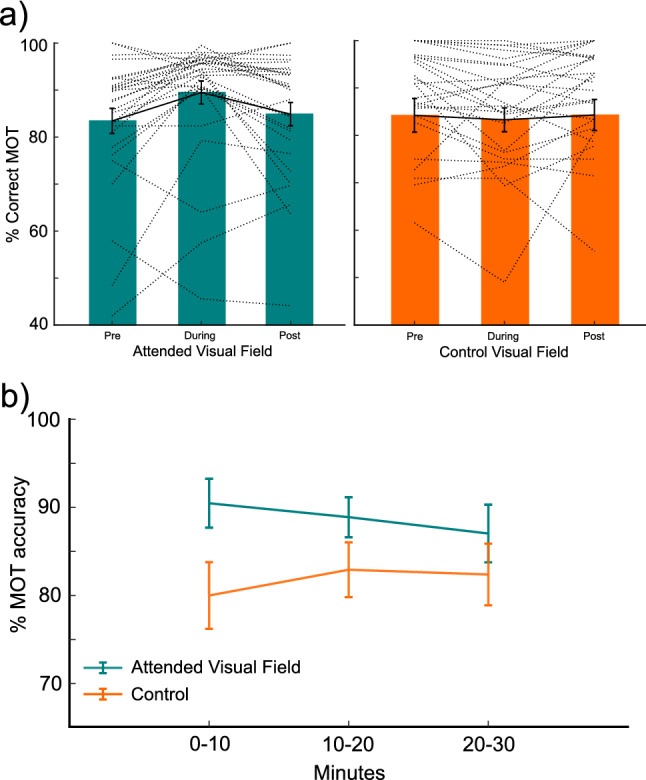
Percentage correct multiple object tracking accuracy pre-, during, and post-manipulation. (**a**) MOT percent accuracy for pre-, during-, and post-manipulation for the attended visual field and control. Dotted lines represent individual subject data. (**b**) Percentage correct multiple object tracking accuracy for each 10-min bin during manipulation period. Attended visual field in teal (unilateral tracking), Control visual field in orange (bilateral tracking).

Importantly, we found no difference between tracked, ignored, and control visual fields prior to manipulation (*p* > 0.05; *emmeans()* with *adjust “mvt”*; Fig. 2a, b; all individual *p* values reported in the Supplemental Table 1). To demonstrate increased attention at the Attended Visual Field during manipulation in comparison to Control, we quantified the change in attention across sessions (Fig. 3a). We found an interaction between Session (pre, during-, or post-manipulation) and Group (Attended Visual Field versus Control; χ^2^(2) = 16.4781, *p* < 0.0003, *glmer*), demonstrating MOT accuracy was modulated as a function of Group and Session. We found a significant increase in accuracy from pre- to during-manipulation for the Attended Visual Field (estimate = 0.734, se = 0.150, z = 4.884, *p* < 0.0001, confidence interval (CI) = 0.344, 1.125; *emmeans()* with *adjust “mvt”*) and a decrease from during- to post-manipulation (estimate = 0.608, se = 0.155, z = 3.915, *p* = 0.0005, confidence interval (CI) = 0.204, 1.012; *emmeans()* with *adjust “mvt”*). As was reported previously, there was no significant difference between pre- and post-manipulation for the Attended Visual Field. There were also no significant differences between sessions in the Control conditions (*p* > 0.05; all individual *p* values reported in the Supplemental Table 2). Comparing the Attended Visual Field with Control during manipulation, we find an increase in tracking performance in the Attended Visual Field (estimate = 0.070, se = 0.032, t = 2.201, *p* = 0.0345, confidence interval (CI) = 0.005, 0.134; *emmeans()*). The increased tracking accuracy from pre- to during-manipulation in the Attended Visual Field only demonstrates attention increase during the manipulation period, when the task was focused within one visual field for 30 min. Furthermore, we split the during-manipulation data into 10-min time-bins to examine how isolation impacted behavior across the 30-min period. We found no main effect of group (χ^2^(1) = 2.8603, *p* = 0.09, *glmer*), nor time-bin (χ^2^(2) = 2.5785, *p* = 0.2748, *glmer*) but we did find an interaction between group and time-bin (χ^2^(2) = 8.8988, *p* = 0.012, *glmer;* Fig. 3b). The interaction was driven by a significant difference within the first 10-min between the Attended Visual Field condition and the Control (estimate = 0.967, se = 0.391, z = 2.469, *p* = 0.0265, confidence interval (CI) = 0.097, 1.84; *emmeans()* with *adjust “mvt”*). This finding indicates the modulation of attention was strongest at the beginning of the manipulation.

### Does attention deterioration in the attended visual field cause the lack of attention change post-manipulation?

**Figure 4:**
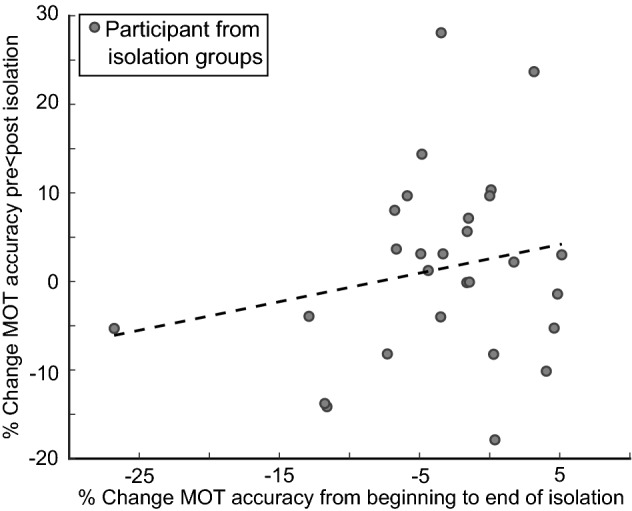
Percentage change in attended visual field from pre- to post-intervention correlated with percentage change in performance from first to last 10 min of training.

We found a significant difference between the unattended and attended visual fields following the attention isolation manipulation. It is plausible the difference was due to attention deterioration following prolonged attention in the Attended Visual Field. Thus, we correlated percentage change pre- to post-manipulation in the Attended Visual Field with percentage change in tracking from the first to last 10 min of the isolation period (Fig. 4). We found no relationship between attention change during the isolation period and the performance change in the Attended Visual Field (r = 0.2048, *p* = 0.2958). The lack of relationship indicates attention deterioration did not drive the absence of change in post-manipulation performance for the Attended Visual Field.

## Discussion

We unbalanced lateralized attention processing via forcing attention to one visual field for a prolonged duration. This manipulation resulted in an increase in attention to the opposite visual field immediately after manipulation. When tracking objects within both visual fields, attention is controlled via balanced excitatory-inhibitory interactions between lateralized sensory processing regions^2^. Balanced neural activity is commonly controlled via homeostatic plasticity which compensates for changes in activity levels to maintain stability of neuronal excitability^19^. After our attentional isolation manipulation, the inactive attention processing regions may benefit from homeostatic gain control in the attempt to rebalance whole visual field attention post-manipulation^20,21^. Attentional gain could be a product of cortical excitability, or a reduction in variability of population neural responses^22,23^.

Alternatively, we might speculate that post-inhibitory rebound spiking could also be the underlying physiological mechanism responsible for increased tracking in the unattended visual field. Post-inhibitory rebound typically occurs following hyperpolarization of the cell membrane^24^. Imaging and noninvasive brain stimulation studies have shown that prolonged lateralized attention recruits unilateral attention processing regions^25^, which in turn inhibit cortical homologues processing in the opposite visual field^2^. It is plausible an increase in tracking performance in the unattended visual field is a result of post-inhibitory spiking, a mechanism which can initiate long term potentiation^26^. Since we recorded the impact of manipulation offline (up to 10 min after), Hebbian plasticity might help explain our post attentional isolation behavioral increase in performance^27^.

Although plausible, the post-inhibitory rebound interpretation of our data contrasts with previous research examining intracortical sensory imbalance, which found reduced inhibition^17^. In adults, imbalance of visual input by covering one eye (monocular deprivation) causes an increase in processing for the *deprived* eye^15^. The strengthening of the deprived eye has parallels to our study, where we find the strengthening of attention to the unattended (or attention-deprived) visual field. Using magnetic resonance spectroscopy (MRS), Lunghi et al.^17^ demonstrated the strengthening of the deprived eye correlated with decreased GABAergic inhibition in the early visual cortex. The lack of inhibition described by Lunghi et al.^15^ indicated that post-inhibitory rebound may not have caused the attention gain found here. However, rebound activity with decreased inhibition has also been demonstrated when examining center-surround receptive fields in cat primary visual cortex^28^, where rebound activity was mediated by lack of excitation of the center rather than increased inhibition from the surround. Reduced GABAergic inhibition following monocular deprivation found by Lunghi et al.^17^ may also explain our lack of change from pre-to post-manipulation in the Attended Visual Field. The post-manipulation facilitation in the Ignored Visual Field did not cause increased homotopic inhibition of the Attended Visual Field. We could speculate that our manipulation also decreased inhibition between lateralized attention areas. However, our results cannot uncover GABAergic concentration changes, but suggest open avenues for future research on the role of inhibition during attention isolation.

Our canonical view of attention is rooted in trade-offs. Inherently, when we attend to one item, color, visual space, or moment in time, other distractors are suppressed, as defined by the normalization model of attention^18^. The trade-off we discuss in this paper is between attention to the left and right visual fields, but with the modulating factor being the loci of attention for the previous 30-min. Trade-offs in attention tend to focus on spatial information^18,29,30^ or short temporal periods (~ 1 s;^31^). However, the history of attention has been demonstrated to be a strong predictor for future attentional performance via priming^32^, training^33^, cognitive fatigue^34^, and sustained attention decline^35,36^. The initial unbalancing of attention during the manipulation period is likely supported by the normalization model of attention. However, the subsequent impact of unbalanced attention on attentional performance tens of minutes following the manipulation has some features which initially seem unexplained by the normalization of attention. Firstly, our finding of increased attention to the previously unattended location. The normalization model of attention does not illustrate a reactive activity increase following suppression. However, the normalization model does not currently consider the history of attention and its subsequent impact on attention. Increased firing following prolonged suppression has been demonstrated in behavior, for example in color saturation afterimages^37^. Secondly, our lack of suppression of the attended visual field as a result of the increased attention in the previously unattended location. According to the normalization model of attention, increased attention to one location should result in suppression of task irrelevant locations. However, in the post-manipulation phase, the subjects are performing bilateral tracking, therefore task relevant information exists in both visual fields. Due to the bilateral nature of the task, suppression of the previously attended visual field would not be supported by the normalization model of attention.

Our results demonstrated that 30 min of tracking in one visual field did not systematically induce training within the attended field, and therefore could not account for a training transfer to the unattended visual field. Previous research has demonstrated multiple sessions of MOT are necessary for successful training^33^. Although there was no systematic training effect in MOT in the Attended Visual Field, we do find large variance in performance, similarly to the Control condition. This variance suggests some subjects experienced a training effect, whilst performance deteriorated for others^36^.

Behavioral gain in the ignored visual field following attention isolation ignites many questions. Firstly, what is the underlying mechanism which supports the increase in tracking ability? Follow-up MRS studies examining GABA and glutamate complex concentration following attention isolation would characterize the role of inhibition in our behavioral finding^38^. A more concrete understanding of underlying mechanisms would support the use of attention isolation as an intervention for unilateral deficits. Secondly, following along the lines of intervention, could isolation be a useful tool for the rehabilitation of patients with unilateral stroke exhibiting lateralized deficits? Exacerbating the existing imbalance in sensory and control processing may cause post-isolation increase favoring contralesional performance. The intervention would focus on the *healthy* hemisphere driving recovery, with a clear benefit in the ease of the approach for stroke survivors. The more successful interventions for unilateral stroke and contralateral attention deficits include tools such as prism goggles^39^ and non-invasive brain stimulation^40,41^. However, the longevity of these approaches is unknown and there is a call for new theory-based rehabilitation methods which can provide relief from attentional bias with user-friendly, at-home approaches^42^. Perhaps with multiple sessions of right attention isolation, we could also cause a long-lasting plastic change improving left visual field attention in right lateralized stroke patients. Interestingly, six 2-h sessions of monocular deprivation to the unhealthy eye can restore visual acuity in amblyopic adults lasting at least 1 year^16^. The restoration of visual acuity has been interpreted as a plastic change following multiple prolonged periods of decreased inhibition between the two eyes during monocular deprivation. Furthermore, for the application of attention isolation as an intervention, future research should focus on the generalizability of the intervention. Here, unbalancing lateralized attention processing and the behavioral outcome of this manipulation was measured using multiple object tracking, a sustained attention task. Ultimately, to apply this intervention successfully in neurological patients, a demonstration of the beneficial effects transferring to everyday activities requiring spatial attention is warranted. Thirdly, is the performance increase following isolation a shared characteristic across other lateralized sensory and control networks? Characterizing a ubiquitous gain following processing imbalance could be useful for promoting interventions across other modalities. Finally, sensory specific attention has been demonstrated to suppress cortical processing of other senses^43,44^, suggesting activity gain for the processing of other senses may be possible using prolonged attention isolation.

There were two limitations to our study. First, due to the novelty of the manipulation and our uncertainty on the expected effect size and associated variance, we did not perform an *a priori* power analysis. We had fourteen participants per group, however we doubled our data points per condition as we collapsed across visual field due to a lack of difference of the manipulation whether it was performed in either the left or the right visual field. We also present our single subject data for transparency. Second, we did not perform the study with a within-subjects design due to concerns of carry-over effects. We have previously found multiple sessions of bilateral multiple object tracking can increase tracking performance which could have interfered with our control condition^36^. Thus, a between-subjects design allowed us to better quantify the effect of the isolation condition.

## Conclusion

Here we demonstrate the history of visual attention impacts subsequent attentional processing. Prolonged attention isolation to one visual field caused a boost in attention in the unattended visual field. The result of imbalanced lateralized attention may be applicable to other lateralized sensory and control processing mechanisms. The behavioral increase following lateralization imbalance can be leveraged as a simple intervention for patients with contralateral deficits following unilateral stroke.

## Method

### Participants

Forty-six right-handed individuals residing in the Cambridge & Boston area of Massachusetts volunteered to take part in the experiment (26 females; age range 20–40 years). The study was approved by Harvard University’s Institutional Review Board: The Human Research Protection Program and the experiment was performed according to their guidelines and regulations. All participants were over the age of 18 and gave written informed consent. Four participants were excluded from analysis due to data recording errors, leaving 42 participants in total. All participants had normal or corrected-to-normal vision.

### Stimuli

Participants viewed the stimuli on a 24-inch LCD Dell screen at a distance of 50 cm (screen resolution: 1980 × 1200), run via a 2010 Apple Mac mini. All stimuli were presented using Psychtoolbox in Matlab^45^.

### Bilateral multiple object tracking paradigm

Bilateral multiple object tracking (MOT) is a well-established task for the recruitment of top-down attention^46^. When participants perform bilateral MOT (in both left and right visual fields simultaneously), lateralized attention processing regions are activated in the right and left hemispheres^25^. In bilateral MOT, each trial began with a fixation point presented centrally for 1000 ms on a gray background (luminance: 19.5 cd/m^2^). Participants were required to maintain central fixation for the duration of the experiment controlled via an Eyelink 1000 Plus. If participants moved their eyes outside of a 1.5° boundary box, the trial was immediately restarted (see *Eye-tracking Acquisition)*. Four objects (black discs, 1.89 cd/m^2^. radius 0.25°) were then presented either side of fixation (eight discs total). Two discs on either side of fixation flashed (at 2 Hz for 2 s) to indicate the targets participants had to track for the duration of the trial. All discs (targets and distractors) then moved along random trajectories within a 6°-by − 6° area, at a constant speed for 4000 ms. The speed of the objects was set according to individual subjects’ threshold (see *Thresholding* section). The closest an object could come to fixation on the left or right visual field was 2°. Each object repelled each other to maintain a minimum of 1.5° space, bouncing off the invisible boundaries, and never crossing the vertical midline. Once the objects stopped moving, one object was highlighted in red on either the left or right of fixation and the participant had to respond if the highlighted object was a “target” or “distractor” with a button press. Importantly, the left and right visual field were tested equally, randomly interleaved in the run. The participant was not aware of which visual field would be tested until the end of the trial, necessitating attention to targets in both visual fields throughout the duration of the task. After the button press, the fixation point turned green to indicate a correct answer, or red to indicate an incorrect answer. Each trial lasted 9.5 s total.

### Unilateral MOT during manipulation

Participants in the manipulation groups performed unilateral MOT during the manipulation period. Whilst fixating centrally and attending to stimuli in one visual field, lateralized attention processing regions are active in the contralateral hemisphere^13^. Group 1) performed right unilateral MOT to isolate attention right and Group 2) performed left unilateral MOT to isolate attention left (Fig. 1b). In unilateral MOT the participants were asked to track two objects in amongst two distractors in only one visual field for the duration of the manipulation while maintaining fixation within 1.5° of fixation (see *Eye-tracking Acquisition*). Four objects were also presented in the untested visual field to equalize visual field sensory input.

### Thresholding MOT speed

At the beginning of the experimental session, each participant performed the bilateral MOT task at different speeds to find the speed at which they performed at 75% correct (Fig. 1b). At 75% correct participants perform below ceiling prior to manipulation, enabling examination of behavioral change due to the experimental manipulation. All subjects began with 16 trials of MOT to practice at the lowest possible speed (2°/sec, the easiest condition, see *Multiple Object Tracking Paradigm (MOT)* section for details). Following practice, seven test blocks were performed using a constant speed approach to thresholding. In each trial of the test block, the speed of the objects was randomly assigned between 2°–16° per second, with 16 trials per block. Therefore, the duration of thresholding was 20 min. Linear interpolation was used to determine the speed at which each individual performed at 75% correct (average threshold speed = 6.71 deg/sec; SD 2.68; Supplemental Fig. 2).

### Procedure

Following recruitment, each participant was randomly assigned to one of three groups. Across the three groups each participant performed the same experimental procedure, except during the manipulation phase (see Fig. 1b). First, participants performed the MOT thresholding task to measure the speed at which each participant performed 75% correct (*see Thresholding MOT speed*). Then participants were required to perform 10 min of bilateral MOT at their fixed individual speed threshold to obtain a pre-manipulation performance baseline measure. Next, participants entered the manipulation phase. Participants in Group 1 isolated attention to the right visual field for 30 min performing unilateral MOT whilst maintaining central fixation (see *Unilateral Multiple Object Tracking Paradigm & Eye-tracking Acquisition*), while participants in Group 2 isolated attention to the left visual field for 30 min in an otherwise identical procedure to Group 1. Therefore, in the manipulation phase, both Groups 1 and 2 tracked two targets in amongst two distractors within one visual field. Finally, participants in Group 3 performed 30 min of bilateral MOT, testing tracking performance in both the left and right visual field equally, thereby experiencing no isolation of attention to one visual field. Therefore, in the manipulation phase, Group 3 tracked two targets in amongst two distractors in each visual field. Immediately after manipulation all participants performed 10 min of post-test bilateral MOT to determine if the manipulation had impacted MOT accuracy. In Group 1 and 2 we were interested in the modulation of attention in the ignored visual field after prolonged attention isolation (Highlighted in maroon in Fig. 1). Group 3 served as a control for any learning effects following prolonged MOT task. Group 3 were tracking more objects per trial during manipulation than Groups 1 and 2 (two in each visual field). This control was selected based on how attentional resources are divided within and between hemifields during visual tracking^47,48^. Our goal was to manipulate visual attention, rather than visual input. Therefore, to keep sensory stimulation the same across conditions, subjects saw the same number of items (four in each visual field) on each trial across all conditions^48^. It is also well established that subjects can track twice as many targets when they appear in separate visual fields compared to when they appear in the same hemifield^47^. Therefore, our control equalized sensory input to the experimental conditions, but attention was across the visual field, rather than isolated to one visual field. On average, subjects performed 61 trials (standard deviation (SD): 6) in the pre-manipulation phase, 167 trials (SD: 28) during manipulation, and 60 trials (SD: 9) post-manipulation. Trial number varied due to participants ability to fixate centrally (see *Eye-tracking Acquisition*).

### Eye-tracking acquisition

An Eyelink 1000 Plus eye-tracking system (SR Research) was used to ensure participants maintained fixation during each trial of the experiment. Calibration was performed at the beginning of each run. During the experiment, an invisible boundary box of 1.5° × 1.5° was placed around the central fixation point, any time a participant moved their eyes outside of the boundary box, the trial restarted. Participants could blink comfortably without restarting the trial.

### Behavioral data analysis

We first fit a general linear mixed effects model on participants multiple object tracking accuracy in R (R Core Team, 2019) using *glmer()* function from the *lme4* package^49^. The between-subject predictor was Manipulation (Attended Visual Field, Ignored Visual Field, or Control) and the within-subject predictors were Session (Pre- or Post- Manipulation) and Visual Field (Left or Right). Following model comparisons, the formula for the selected model was: *glmer (Accuracy* ~ Manipulation * Session * VF + *(1* + *Session|Subs)* + *(1* + *VF|Subs), data* = *AttIso, family* = *binomial)*. The linear mixed effects model was trial based and random intercepts and slopes were included for each participant. We performed model comparisons to determine which interactions best fit the data. *Chi-squared* and p-values were reported for the interactions and main effects. Individual contrasts were performed using *emmeans()* and *adjust* = *”mvt”* for multiple comparisons^50^. Analysis script and data are available for download and review at: https://osf.io/3kus7/?view_only=4322dca7c5a64276a561acccfc9e3400.

## Supplementary Information

Supplementary Information.

## Data Availability

Data and analysis scripts are available at the following link: https://osf.io/3kus7/?view_only=4322dca7c5a64276a561acccfc9e3400.
