# Supplementary material for: Behavioral gain following isolation of attention
Source: Sci Rep. 2021 Sep 29;11:19329. doi: 10.1038/s41598-021-98670-w (PMC8481494; doi:10.1038/s41598-021-98670-w)
Supplement: Supplementary file 1 — Supplementary Information. [file 41598_2021_98670_MOESM1_ESM.pdf]

Supplemental Information

Behavioral gain following isolation of attention

Grace Edwards, Anna Berestova, Lorella Battelli

Model Comparisons

Model comparisons were conducted on various *glmer* models to determine the best model fit for the data collected.

Syntax:

```
Mod1 <- glmer (Accuracy ~ Manipulation + Session + VF + (1+Session|Subs) + (1+VF|Subs), data = AttIso, family = binomial)
Mod2 <- glmer (Accuracy ~ Manipulation * Session + VF + (1+Session|Subs) + (1+VF|Subs), data = AttIso, family = binomial)
Mod3 <- glmer (Accuracy ~ Manipulation + Session * VF + (1+Session|Subs) + (1+VF|Subs), data = AttIso, family = binomial)
Mod4 <- glmer (Accuracy ~ Session + Manipulation * VF + (1+Session|Subs) + (1+VF|Subs), data = AttIso, family = binomial)
Mod5 <- glmer (Accuracy ~ Manipulation * Session * VF + (1+Session|Subs) + (1+VF|Subs), data = AttIso, family = binomial)
```

anova(Mod1, Mod2, Mod3, Mod4, Mod5)

Mod	Df	AIC	BIC	loglik	deviance	Chisq	Chi Df	Pr(>Chisq)
Mod1	11	3540.1	3611.1	-1759.1	3518.1			
Mod3	12	3541.9	3619.3	-1759.0	3517.9	0.2183	1	0.64350
Mod2	13	3534.7	3618.5	-1754.3	3508.7	9.2373	1	0.002371
Mod4	13	3539.8	3623.7	-1756.9	3513.8	0	0	1.00000
Mod5	18	3538.5	3654.6	-1751.3	3502.5	11.33005	5	0.045738

Model comparisons demonstrated Mod2 is the least complex and best fit for our data.

**Contrast**	**Estimate**	**SE**	**z.ratio**	**p.value**
Control > Ignored	0.482	0.356	1.356	0.3451
Ignored > Attended	-0.233	0.137	-1.694	0.1922
Attended > Control	-0.250	0.357	-0.700	0.7512
Control LVF > Ignored LVF	0.605	0.438	1.381	0.3505
Control LVF > Attended LVF	0.841	0.436	1.929	0.1303
Ignored LVF > Attended LVF	0.236	0.410	0.575	0.8336
Control RVF > Ignored RVF	0.450	0.418	1.077	0.5283
Control RVF > Attended RVF	-0.244	0.423	-1.576	0.8329
Ignored RVF > Attended RVF	-0.694	0.414	-1.677	0.2138

Supplemental Table 1: Comparisons between groups pre-manipulation. Individual statistics for each comparison.

Contrast	Estimate	SE	z.ratio	p.value
Control Dur > Control Pre	-0.711	0.190	-0.902	0.8690
Control Dur > Control Post	-0.1446	0.193	-0.748	0.9292
Control Pre > Control Post	0.0265	0.182	0.146	0.9999

Supplemental Table 2: Comparisons between sessions in the control condition. Individual statistics for each comparison.

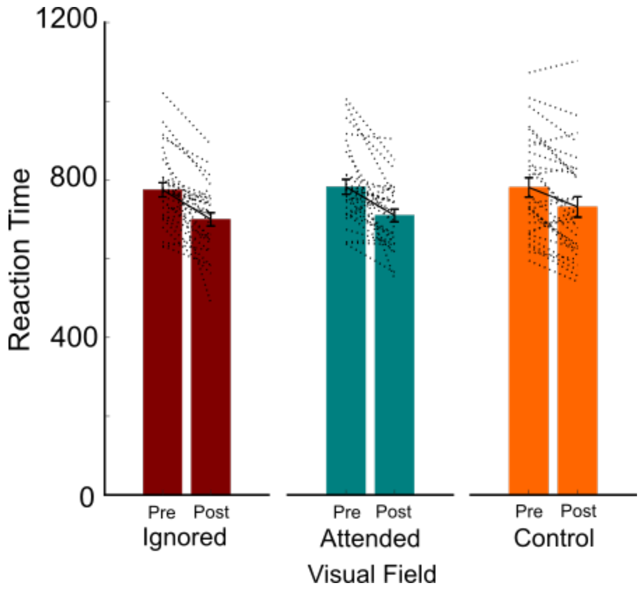

Supplemental Figure 1: Reaction time in MOT task for pre- and post-manipulation. Ignored visual field in maroon, attended visual field in teal, control visual field with bilateral tracking in orange.

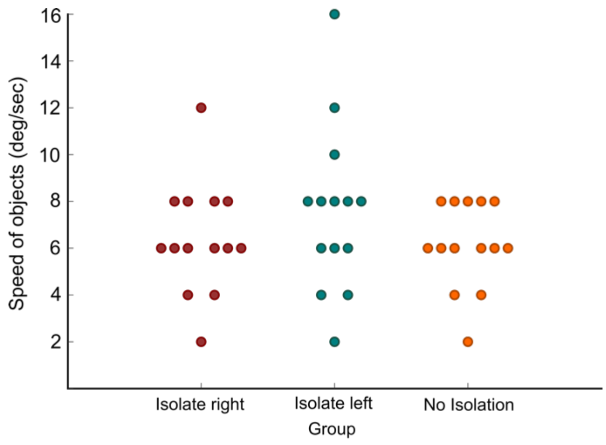

Supplemental Figure 2: Thresholding value per participant per group.

One-way ANOVA determined no difference in thresholding speed between the three groups (F(2,39)1.12, p=0.337).
